# The Pathophysiology of Cardiac Abnormalities in Cantu Syndrome: Perspective on “The Mechanism of High-Output Cardiac Hypertrophy Arising From Potassium Channel Gain-of-Function in Cantú Syndrome”

**DOI:** 10.1093/function/zqaa005

**Published:** 2020-06-23

**Authors:** Qadeer Aziz, Andrew Tinker

**Affiliations:** William Harvey Heart Centre, Barts and The London School of Medicine and Dentistry, Queen Mary University of London, Charterhouse Square, London EC1M 6BQ. UK; William Harvey Heart Centre, Barts and The London School of Medicine and Dentistry, Queen Mary University of London, Charterhouse Square, London EC1M 6BQ. UK

Cantu syndrome (CS) is a rare human disease first described in the early 1980s.^1^ It is a complex disorder characterized by hypertrichosis, distinct skeletal abnormalities and syndromic features such as coarse facial features. There are an array of cardiovascular abnormalities including patent ductus arteriosus, pericardial effusions, pulmonary hypertension, low blood pressure, dilated and tortuous blood vessels, and pronounced cardiac hypertrophy.^2^ Genetic analysis using exome sequencing of CS patients has recently revealed that CS is caused by spontaneous dominant mutations in subunits that constitute the ATP-sensitive potassium (K_ATP_) channel. Specifically, these are gain-of-function (GoF) mutations in *ABCC9* (regulatory sulfonylurea receptor subunit [SUR2]) and less commonly in *KCNJ8* (pore-forming subunit, Kir6.1). K_ATP_ channels are widely expressed in a range of tissues throughout the body. Their ability to couple intracellular metabolism to membrane excitability allows K_ATP_ channels to regulate diverse functions such as insulin release, neuro- and cardioprotection, and blood flow.^^2–5^^

A functional K_ATP_ channel exists as a hetero-octomeric complex composed of four pore-forming potassium channel subunits (either Kir6.1 or Kir6.2) and four regulatory sulfonylurea receptor subunits (either SUR1, SUR2A, or SUR2B). These subunits can come together to form tissue-specific combinations giving rise to K_ATP_ channels with heterogenous properties, cellular roles, and distinct pharmacological profiles. For example, K_ATP_ channels in vascular smooth muscle are formed of Kir6.1 (*KCNJ8*) and SUR2B (a splice variant arising from *ABCC9*) and regulate smooth muscle tone and blood pressure.^3^^,^^4^ All known CS mutations in Kir6.1 and SUR2 lead to increased activity in recombinant K_ATP_ channels when expressed in heterologous expression systems.^2^^,^^6^ The Nichols group has used a CRISPR/Cas9 gene-editing approach to produce mouse models with CS-causing mutations in *Kcnj8* (Kir6.1) and *Abcc9* (SUR2). Patch-clamp studies of vascular smooth muscle cells show increased K_ATP_ channel activity. The cardiovascular abnormalities seen in CS patients were recapitulated in the K_ATP_ GoF mice. CS mice presented with dilated blood vessels, low blood pressure, reduced systemic vascular resistance and marked cardiac hypertrophy.^7^ Vasodilation and low blood pressure are expected from over-activity of K_ATP_ channels in vascular smooth muscle as result of hyperpolarization and reduced excitability. However, cardiac hypertrophy is likely secondary to K_ATP_ GoF in vascular smooth muscle as Kir6.2 not Kir6.1 is the predominant pore-forming subunit in the heart.^7^ It is worth noting this isn’t the case with *ABCC9* mutations as SUR2A is the major SUR in the heart and earlier studies did show increased L-type calcium current in myocytes.^8^

In this issue of *FUNCTION*, McClenaghan et al., elucidate a potential mechanism leading to the marked cardiac hypertrophy apparent in CS mice.^9^ They show hypertrophy results from enhanced renin–angiotensin signaling (RAS) in response to the chronically low systemic vascular resistance and likely reduced renal perfusion detected by the juxtaglomerular apparatus in the afferent arteriole. RAS increases blood volume through the actions of aldosterone leading to increased cardiac output and angiotensin II is a direct vasoconstrictor counteracting the fall in blood pressure. However, angiotensin II also has direct actions on the heart and chronic elevation may directly promote cardiac hypertrophy. The authors go further and show intriguingly that the typical structural/functional changes associated with “conventional” cardiac hypertrophy and myopathy such as fibrosis, reduced ejection fraction, and increased expression of TGFβ1 are not apparent in CS mouse hearts although RNA-Seq data show altered expression of a myriad of genes associated with pathological hypertrophy. Furthermore, despite this and the pronounced cardiac hypertrophy, basal cardiac function is relatively normal in CS mouse hearts. This remains the case even when the mice are aged to a year and without additional stressors they do not develop overt heart failure. However, there are consequences of a persistent high output state, namely a reduced cardiac reserve and this leads to an impaired exercise tolerance in the mice.

There remain many intriguing questions raised by this work. For example, it is not clear if a similar mechanism is operative in the patients and whether there are long-term clinical sequelae. These studies may catalyze more detailed clinical investigations in this rare patient cohort. The authors suggest that the mechanism is similar in CS mice with *Kcnj8* and *Abcc9* mutations but it remains possible that there may be a direct cardiac effect with the latter and differences in phenotype in the two murine lines. Kir6.1 also underlies currents in endothelial cells and unregulated increased channel activity may also contribute to the vascular pathology.^10^ Finally, the authors venture that the mice may more generally be a model for high output cardiac failure. This entity is classically described with severe thiamine deficiency (“wet beri-beri”) but may also occur in anemia, arteriovenous shunting, thyrotoxicosis, etc. This is a speculative proposal but the gene expression profile elucidated in the ventricle with RNA-Seq gives a molecular signature that could be investigated in other models.

These developments in understanding the underlying mechanism for CS abnormalities may pave the way for a more targeted therapeutic approach. The authors have previously shown that glibenclamide, an inhibitor of K_ATP_ channels used more widely in the treatment of Type II diabetes, has promising effects on the cardiovascular abnormalities occurring in CS reversing cardiac hypertrophy and increasing blood pressure in the murine models.^7^ Glibenclamide may also potentially reverse hypertrichosis since the K_ATP_ channel opener minoxidil is used to treat alopecia. Thus the unpicking of mechanism using these murine models may enable the repurposing of drugs to treat this rare condition.

## Acknowledgements

This work was supported by the British Heart Foundation (RG/15/15/31742) and was facilitated by the NIHR Biomedical Research Centre at Barts.

## Conflict of interest statement

The authors have no conflicts of interest to declare.
